# Biliary Atresia With Extrahepatic Cyst: A Diagnostic Dilemma

**DOI:** 10.7759/cureus.17447

**Published:** 2021-08-25

**Authors:** Saru Kunwar, Bom B.C., Ravindra K Sah

**Affiliations:** 1 Pediatrics, Ishan Children and Women's Hospital, Kathmandu, NPL; 2 Radiology, Rapti Academy of Health Sciences, Dang, NPL; 3 Pediatrics, Arya Care and Cure Hospital, Janakpur, NPL

**Keywords:** biliary atresia, choledochal cyst, cholestasis, cystic biliary atresia, extrahepatic cyst, intracranial hemorrhage

## Abstract

Biliary atresia (BA) is a rare disorder that usually presents with cholestatic symptoms such as jaundice, pale stool, and dark urine shortly after birth. Intracranial hemorrhage is a rare manifestation. BA may sometimes be associated with a cyst in the biliary tree. Differentiating choledochal cyst with BA or cystic biliary atresia can be quite challenging yet very important as they differ significantly in their prognosis and treatment. Furthermore, clear mucoid fluid in the extrahepatic cyst is an unusual occurrence. This case report aims to raise awareness about this rare variant to facilitate appropriate and timely diagnosis and treatment.

We report a case of an infant who presented with intracranial hemorrhage and conjugated hyperbilirubinemia. The ultrasonographic and magnetic resonance cholangiopancreatography (MRCP) findings suggested Type I choledochal cyst, but it was later diagnosed as biliary atresia with an extrahepatic cyst and was not corrected by Kasai’s operation.

## Introduction

Biliary atresia (BA) is a rare disorder with marked variation in incidence ranging from one in 17,000 to 19,000 live births in the United Kingdom and France and most common in East Asian countries, about one in 5000 in Taiwan. According to the level of most proximal biliary obstruction, it is classified into Type I, II, and III. Type I constitutes 5% of cases with luminal patency up to the common bile duct and proximal part of the cystic duct. Type II constitutes 2% of cases with patency up to the common hepatic duct, and more than 90% of cases are Type III in which the most proximal part of the extrahepatic biliary tract (within the porta hepatis) is entirely solidified [1].

BA mainly presents with conjugated hyperbilirubinemia and persistent alcoholic stool. Hemorrhage may occur, but intracranial hemorrhage as presenting symptoms is rare [2]. When BA presents with a cyst, the main differential diagnoses are cystic biliary atresia or choledochal cyst with BA. However, mucoid content of extrahepatic biliary cyst along with BA is an unusual occurrence. Nevertheless, differentiating them is very important as their prognosis and treatment differ significantly [3-5].

## Case presentation

We present a 30-day-old exclusively breastfed female baby who was brought to our outpatient department with complaints of scanty reddish spots over bilateral upper limbs, excessive cry, fever, yellowish discoloration of eyes, clay-colored stool, along with one episode of seizure. The baby was born to a 30-year-old primigravida mother via spontaneous vaginal delivery at term with an uneventful antenatal and perinatal period. On presentation, the baby was febrile, icteric with scanty purpuric and ecchymotic rashes over bilateral upper limbs. No obvious findings were present on systemic examination.

Laboratory examination (details in Table 1) revealed conjugated hyperbilirubinemia with a deranged coagulation profile. Serum glutamic oxaloacetic transaminase (SGOT) and serum glutamic pyruvic transaminase (SGPT) values were within normal limits, but alkaline phosphatase (ALP) was raised. Serology for TORCH (toxoplasmosis, other agents, rubella, cytomegalovirus, and herpes simplex) infection, HIV (human immunodeficiency virus), and hepatitis B and C was non-reactive; cranial sonogram revealed intraparenchymal hemorrhage, and no abnormality was detected on abdominal ultrasonography. Cerebrospinal fluid analysis was suggestive of meningitis and was treated with intravenous antibiotics.

**Table 1:** Laboratory parameters before and after surgery for biliary atresia ALP, alkaline phosphatase; APTT, activated partial thromboplastin time; E, eosinophils; FFP, fresh frozen plasma; GGT, gamma-glutamyl transferase; Hb, hemoglobin; INR, international normalized ratio; L, lymphocytes; N, neutrophils; POD, postoperative day; PT, prothrombin time; SGOT, serum glutamic oxaloacetic transaminase; SGPT, serum glutamic pyruvic transaminase; UDCA, ursodeoxycholic acid; USG ultrasonography; WBC, white blood cells.

Investigations	Admission	First discharge	One week follow-up	Before surgery (before FFP and blood transfusion)	After surgery	10 days POD	24 days POD	36 days POD	42 days POD + UDCA	49 days POD + Vitamin K injection
Hb (gm/dL)	8	8.9			11.7			12.5		
Platelets/cm^3^	3,47,000				2,20,000			1,78,000		
Total bilirubin (mg/dL)	8.4	16.0	11.2	14.2	10.3	4.2	2.3	4.0	6.9	9.6
Direct bilirubin (mg/dL)	4.2	10	7.8	11.2	7.2	2.6	1.1	2.4	4.5	7.24
SGPT (IU/L)	35	102	165	190	110	290	245	103	88	98
SGOT (IU/L)	40	160	205	245	140	225	230	180	114	119
ALP (U/L)	910	1020	1328	1184	528		1054	1596	625	662
Total protein (g/dL)	9.8	5.9	7.1	6.8			6.8	5.6	6.8	6.4
PT (sec)	15	14		33	16		17	18	15	12
INR	1.25	1.16		2.75	1.33		1.41	1.5	1.25	0.92
APTT (sec)	49	43			44		43	56	60	35
Others	WBC 10,900/mm^3^, N60%, L35%			GGT 569 U/L, albumin 3.9 g/dL		Albumin 4 g/dL		USG color Doppler: normal; WBC: 6,100, N 30%, L 65%, E 3%		Albumin: 3.6 g/dL; albumin globulin ratio: 1.29

Conservative management was done for the intracranial hemorrhage as the child’s general condition was improving significantly. She was then discharged on oral ursodeoxycholic acid and was followed up two weeks later.

On her follow-up visit, ultrasonography (USG) of brain and ventricles was normal; however, USG abdomen showed a cystic structure measuring 13 mm in diameter anterior to the portal vein separate from the gallbladder and was continuous with the common bile duct, findings suggestive of choledochal cyst Type I. The liver function tests and coagulation parameters were deteriorating progressively (Table 1).

**Figure 1:**
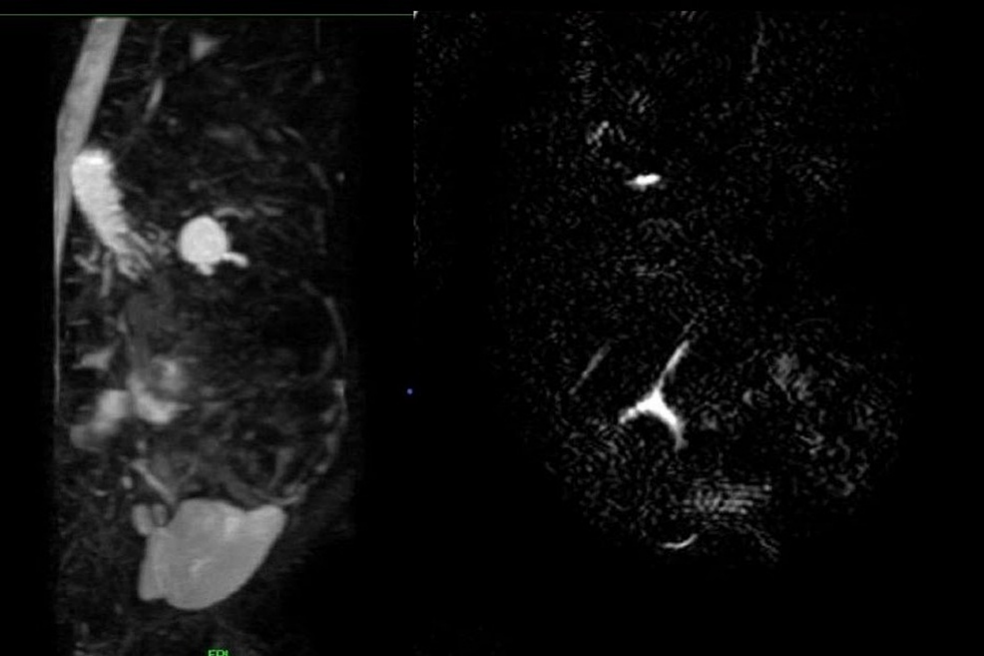
Magnetic resonance cholangiopancreatography (MRCP) image showing cystic lesion in the hepatic hilar region with right and left hepatic duct and a part of common bile duct

Plain magnetic resonance cholangiopancreatography (MRCP) was done, which showed approximately 14 mm × 13 mm-sized cystic lesion in hepatic hilar region in the location of common hepatic duct communicating with the intrahepatic bile ducts, likely Type I choledochal cyst (Figure 1).

**Figure 2:**
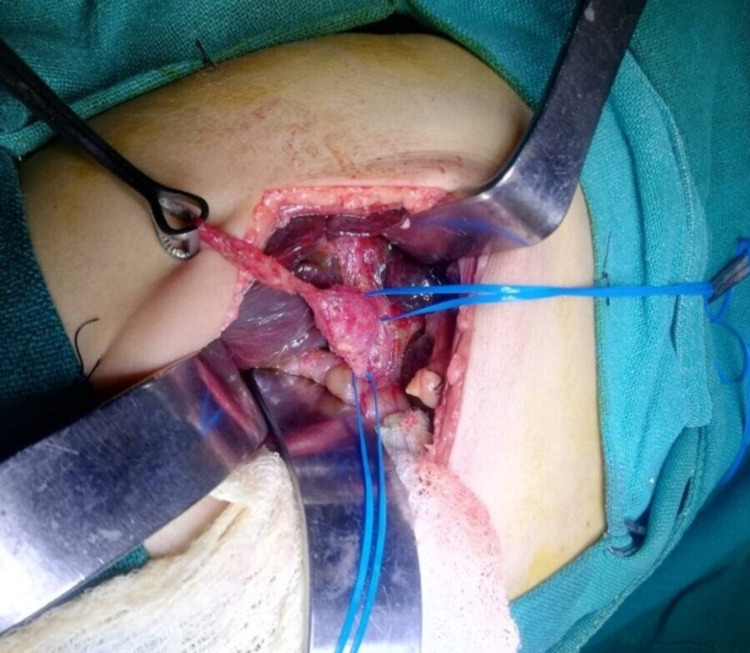
Intraoperative finding showing atretic gallbladder, cystic duct, and common hepatic duct with cystic dilatation at the confluence of these three ducts

Surgical excision of the cyst was planned, but intraoperative findings were consistent with BA Type III with cystic dilatation at the confluence of atretic common hepatic duct, cystic duct, and common bile duct (Figure 2). The cyst contained mucoid fluid and did not communicate with the ducts. Then, Roux-en-Y hepatic portojejunostomy (Kasai’s procedure) was performed.

Histopathological examination reports later confirmed the intraoperative findings. Liver tissue biopsy showed fatty changes and ballooning degeneration of hepatocytes, periportal collection of lymphocytes, and increased fibrocollagen tissue in the portal region, consistent with early cirrhotic changes; tissue from porta hepatis (mucoid/cystic) biopsy section showed biliary canaliculi with ill-defined mucosal lining, extensive fibrocollagen tissue in the stroma, hemorrhagic areas and focal collection of round cells, consistent with BA; gallbladder and common hepatic duct biopsy section showed ill-defined mucosa and extensive hemorrhagic areas.

The child’s symptoms and laboratory parameters improved significantly on subsequent follow-up visits, but one month after the operation, the child started developing pale stools again. Details of the laboratory reports on follow-up visits are given in Table 1. She is currently under treatment with ursodeoxycholic acid, vitamin supplements, and prophylactic antibiotics, and liver transplantation is being discussed as the long-term plan.

## Discussion

Typically, BA presents with jaundice, pale stools, and dark urine shortly after birth [1]. Intracranial bleeding, in particular, occurs at a lower rate of 1%-6% per year in patients with BA in Japan [2]. The reference values for 30-day-old infants are prothrombin time (PT) of 10 to 14.3 seconds, international normalized ratio (INR) of 0.53 to 1.26, and activated partial thromboplastin time (APTT) of 32 to 55.2 seconds [6]. However, in our case, the coagulation parameters were within normal range, although the child had bleeding manifestations. Shearer reports that reliance on PT and/or APTT alone could lead to both false inclusions/exclusions [6].

Abdominal ultrasound is the first-line imaging modality in neonatal cholestasis. The triangular cord sign and gallbladder abnormalities in ultrasonography (USG) have a specificity of 97% and 92%, respectively, for BA diagnosis [7]. USG at the time of presentation in our case was normal, but on a follow-up visit, the findings suggested Type I choledochal cyst. This further demonstrates the need for a follow-up scan in case of persistent conjugated hyperbilirubinemia with normal initial USG. In the context of this USG finding, MRCP was done in our case. A meta-analysis by Wang et al. showed that MRCP has a specificity of 58% and a negative predictive value of 94.3% in diagnosing BA [8]. However, MRCP findings in our case were suggestive of Type I choledochal cyst. An intraoperative cholangiogram is a gold standard for the diagnosis of biliary atresia [7].

Liver biopsy plays a crucial role in the diagnostic work-up of infants with neonatal cholestasis, and histopathological examination of portal tracts holds the key findings in diagnosing BA. Duct/ductal bile plugs, fibroblasts proliferation, and a variable amount of inflammatory cells, especially neutrophils, are usually present [9]. In 5%-10% of infants with biliary atresia, injury to the biliary tree may produce cystic dilation called cystic biliary atresia (CBA) resembling choledochal cyst (CC) [9,10]. CBA and CC are distinct entities histologically but may occasionally overlap. CBA typically lacks epithelial lining and inflammation. The cyst walls have an inner subepithelial sclerotic layer that tends to delaminate, forming grossly visible inner cyst lining and is associated with myofibroblastic hyperplasia. Extrahepatic atresia usually involves both proximal and distal to the cyst. CC, on the other hand, has mostly preserved and uninjured epithelium and no subepithelial sclerotic layer. A smooth muscle layer is present to some extent in CC but is absent in CBA. Management of the two also differs from each other; CBA is managed by Kasai hepatoportoenterostomy, whereas CC is treated by complete cyst excision followed by restoration of biliary enteric continuity when necessary [10].

In our case, BA Type III with non-communicating extrahepatic cyst containing mucoid fluid was present. Two similar cases of BA associated with true cystic structure at the common hepatic or common bile duct were reported by Sookpotarom and Vejchapipat, and Kasai's operation was successful in treating the conditions. The author also reports that awareness of this type of biliary atresia may facilitate the surgeon’s decision during the dissection of the hepatoduodenal ligament [3].

Obaidah et al. reported a case with a pre-operative magnetic resonance imaging (MRI) diagnosis of Type I CC with a blind-ending distal common bile duct [4]. The gall bladder was also clearly visualized in the initial sonogram (similar to our finding), which is unusual for BA. Intra-operatively, a cyst in the subhepatic region was found that communicated with the gall bladder, and aspiration yielded only mucinous fluid and no bile. Complete excision of the cyst along with hepaticojejunostomy did not correct the hyperbilirubinemia, and upon re-exploration, BA Type III was revealed [4]. Similarly, Berkowitz et al. reported a case clinically and radiologically suggesting CC, containing clear fluid on aspiration, but histologically more consistent with CBA [5].

Thus, pediatricians, surgeons, radiologists, and pathologists should be aware of this rare variant to ensure that the condition is not missed and appropriate treatment is given.

## Conclusions

Clear mucoid fluid in the extrahepatic cyst associated with BA is an unusual finding. Differentiating CC with BA or CBA can be challenging clinically, radiologically, and histopathologically. Considering the differences in treatment and prognosis, we should be aware of this rare occurrence and the differential diagnoses.
